# Repetitive Transcranial Magnetic Stimulation for Tinnitus Treatment in Vestibular Schwannoma: A Pilot Study

**DOI:** 10.3389/fneur.2021.646014

**Published:** 2021-04-12

**Authors:** Maria Teresa Leao, Kathrin Machetanz, Joey Sandritter, Marina Liebsch, Andreas Stengel, Marcos Tatagiba, Georgios Naros

**Affiliations:** ^1^Department of Neurosurgery and Neurotechnology, Eberhard Karls University, Tuebingen, Germany; ^2^Section Psychooncology, Comprehensive Cancer Center Tuebingen-Stuttgart, University Hospital Tuebingen, Tuebingen, Germany; ^3^Department of Psychosomatic Medicine and Psychotherapy, University Hospital Tuebingen, Tuebingen, Germany; ^4^Charité Center for Internal Medicine and Dermatology, Department for Psychosomatic Medicine, Charité-Universitätsmedizin Berlin, Corporate Member of Freie Universität Berlin, Humboldt-Universität zu Berlin and Berlin Institute of Health, Berlin, Germany

**Keywords:** repetitive transcranial magnetic simulation, tinnitus, vestibular schwannoma, neurosurgery, neuromodulation

## Abstract

**Background:** Vestibular schwannomas (VS) are brain tumors affecting the vestibulocochlear nerve. Thus, VS patients suffer from tinnitus (TN). While the pathophysiology is mainly unclear, there is an increasing interest in repetitive transcranial magnetic stimulation (rTMS) for TN treatment. However, the results have been divergent. In addition to the methodological aspects, the heterogeneity of the patients might affect the outcome. Yet, there is no study evaluating rTMS exclusively in VS-associated tinnitus. Thus, the present pilot study evaluates low-frequency rTMS to the right dorsolateral pre-frontal cortex (DLPFC) in a VS-associated tinnitus.

**Methods:** This prospective pilot study enrolled nine patients with a monoaural VS-associated tinnitus ipsilateral to the tumor. Patients were treated with a 10-day rTMS regime (1 Hz, 100% RMT, 1,200 pulses, right DLPFC). The primary endpoint of the study was the reduction of TN distress (according to the Tinnitus Handicap Inventory, THI). The secondary endpoint was a reduction of TN intensity (according to the Tinnitus Matching Test, TMT) and the evaluation of factors predicting tinnitus outcome (i.e., hearing impairment, TN duration, type of tinnitus).

**Results:** No complications or side effects occurred. There was one drop-out due to a non-responsiveness of the complaint. There was a significant acute effect of rTMS on the THI and TMT. However, there was no significant long-term effect after 4 weeks. While the THI failed to detect any clinically relevant acute effect of rTMS in 56% of the patients, TMT revealed a reduction of TN intensity for more than 20 in 89% and for more than 50 in 56% of the patients. Notably, the acute effect of rTMS was influenced by the TN type and duration. In general, patients with a tonal TN and shorter TN duration showed a better response to the rTMS therapy.

**Conclusion:** The present pilot study is the first one to exclusively evaluate the effect of low-frequency rTMS to the right DLPFC in a VS-associated tinnitus. Our results prove the feasibility and the efficacy of rTMS in this patient cohort. There is a significant acute but a limited long-term effect. In addition, there is evidence that patients with a tonal tinnitus and shorter tinnitus duration might have the strongest benefit. A larger, randomized controlled study is necessary to prove these initial findings.

## Introduction

Brain tumors affect functions related to the affected neuronal structure. Hence, patients with a vestibular schwannoma (VS), a benign tumor of the vestibulocochlear nerve, suffer from audiovestibular symptoms (i.e., hearing loss, tinnitus, or dizziness) (1–4). Tinnitus (TN) is affecting 63–75% of the VS patients (3, 5–7) and significantly impairing patients' quality of life (2, 8, 9). Notably, VS-associated tinnitus, however, is one of the few TN conditions accessible to causal therapy (10, 11). Thus, TN ceases in one third of the patients after a surgical removal of the tumor (4, 12, 13). The pathophysiology of the VS-associated TN has not yet been fully clarified (3, 4, 10). However, it is generally assumed that the pathophysiology might be similar to that of idiopathic TN (3). The current concept hypothesizes spurious auditory signals after partial sensory deafferentation, e.g., after damage to the cochlea (e.g., bang trauma) or cochlear nerve (e.g., vestibular schwannoma), to cause TN onset (1, 14–17). After chronification, however, TN perpetuation is theorized to depend on central maladaptive neuroplasticity because of the disturbed signal-to-noise ratio. These neuroplastic changes are thought to cause a neuronal hyperexcitability for the residual auditory input resulting in the subjective misperception (10, 11, 18, 19). Having a central origin, TN is hardly accessible to therapy (20). In the last years, there is a growing interest in using repetitive transcranial magnetic stimulation (rTMS) in TN therapy (20–24). rTMS is suggested to modify the excitability of relevant neurons and neurotransmitter systems in TN (25). However, the results have been divergent and even contradictory (21, 24, 26–29). First, there are still unsolved methodological issues, e.g., uncertainties concerning appropriate stimulation sites and stimulation intensities (20, 23). Second, there has been a large heterogeneity in the treated patient cohorts (20, 26). Most studies have evaluated the rTMS effects in patients with an idiopathic TN which are characterized by a large variability or ambiguity of the underlying cause. Additionally, the amount of hearing loss, tinnitus duration, and quality of the tinnitus seem to play important roles for the treatment outcome (26, 30, 31). In contrast, patients with anatomical causes of TN, such as VS, are usually excluded from rTMS studies. To our opinion, however, these patients represent a relatively homogenous cohort with a definite TN origin (3, 4). As these patients are presumably seeking medical advice in an early stage of the disease, the TN duration will be shorter, which is beneficial for rTMS treatment (32, 33). Yet, there is no rTMS study explicitly analyzing patients with a VS-associated TN.

The aim of the present pilot study was to provide the first evidence for the feasibility and effectivity of low-frequency rTMS to the right DLPFC in a VS-associated TN. Here, we describe our first experience in nine patients indicating an acute effect or rTMS on TN perception as measured by questionnaires and TN matching.

## Methods

### Patients

**Figure 1:**
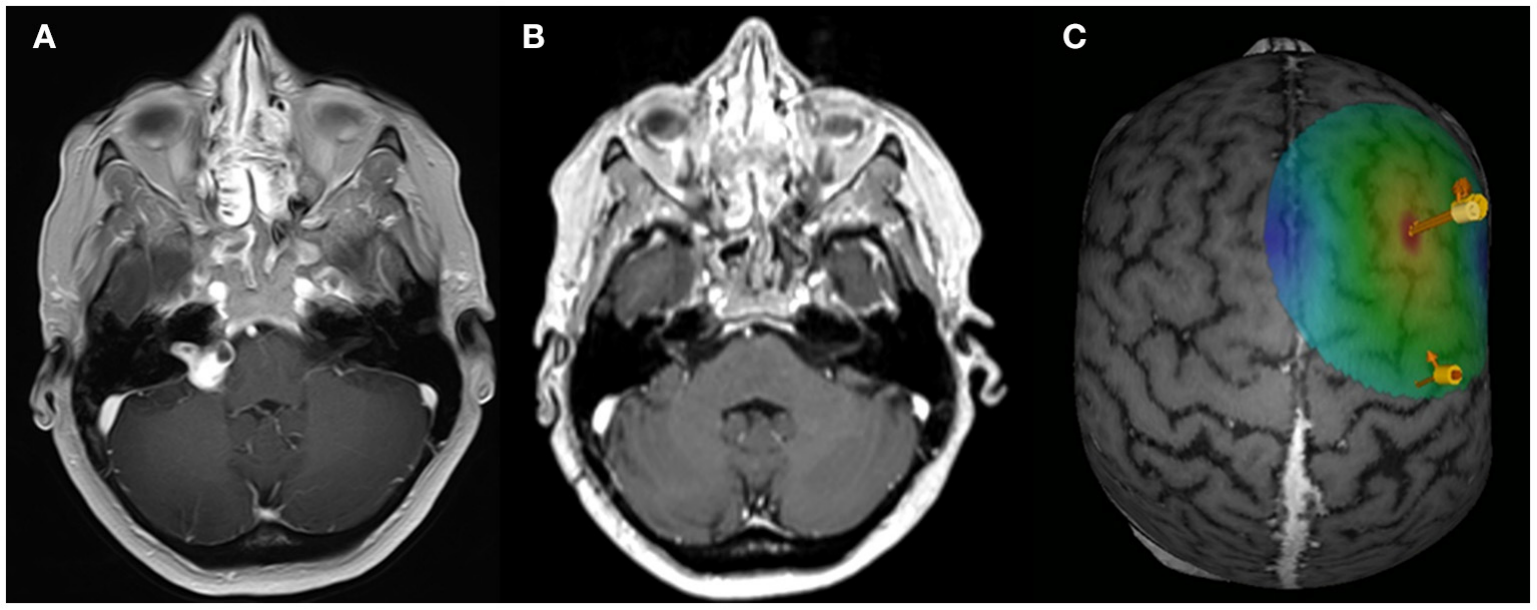
rTMS paradigm. Exemplary axial MRI data (contrast-enhanced T1 sequence) of patient ID1 with a right-sided VS for the preoperative **(A)** and post-operative **(B)** situation. **(C)** Exemplary data of the rTMS application site. Arrows are indicating the APB hotspot and the rTMS on the DLPFC.

This prospective study enrolled nine patients (57.1 ± 10.6, four female) with a unilateral sporadic VS who were treated at the Neurosurgical Department of the University of Tuebingen, Germany (Figures 1A,B). The inclusion criteria covered an age range of 18–80 years old and the presence of a monoaural TN ipsilateral to the tumor. Exclusion criteria were pregnancy, contralesional hearing impairment, and the presence of additional neurological conditions (e.g., epilepsy). Patient characteristics are shown in Table 1. Patients gave a written informed consent to their participation. This study was approved by the ethics committee of the Eberhard Karls University Tuebingen and performed in accordance to the Declaration of Helsinki.

**Table 1:** Data overview.

**ID**	**1**	**2**	**3**	4^*^	**5**	**6**	**7**	**8**	**9**	**Sum**
**Age**	60.0	51.3	67.6	71.6	44.3	33.6	60.3	47.5	67.4	57.1 ± 10.6
Gender	F	M	F	M	M	M	f	M	F	4:5
**Tumor**										
Size	T3	T3	T2	T2	T3	T3	T2	T2	T3	4:5
Side	Right	Left	Left	Right	Left	Right	Left	Left	Left	4:5
**Hearing**										
Ipsilateral	4	5	4	3	5	1	1	3	5	3.4 ± 1.6
Contralateral	1	1	1	1	1	1	1	1	1	
**Tinnitus**										
Type	T	N	T	N	N	T	N	N	T	4:5
Side	Right	Left	Left	Right	Left	Right	Left	Left	Left	4:5
Onset	Pre	Post	Post	Pre	Post	Post	Pre	Pre	Pre	5:4
Duration (y)	12.6	4.0	2.9	12.0	1.3	0.9	0.9	3.3	2.3	4.5 ± 4.6
**rTMS**										
Intensity (%)	38	36	50	30	45	29	32	40	30	37 ± 7
**TMT**										
Pre Freq (kHz)	7.0	–	3.0	–	–	5.4	–	–	4.0	4.8 ± 1.7
Int (dB)	40	34	64	50	44	35	30	45	33	42 ± 11
Post Freq (kHz)	5.8	–	2.5	–	–	1.5	–	–	3.6	3.3 ± 1.3
Rel. int	0.39	0.67	0.1	1.0	0.43	0.05	0.25	0.53	0.20	
**THI**										
Pre	4	44	54	98	52	38	20	34	32	42.0 ± 26.0
Post	2	36	40	–	52	22	18	32	22	28.0 ± 15.3
FU	2	46	50	–	52	34	24	34	30	34.5 ± 16.9
**HHI**										
Pre	8	18	60	18	36	8	10	18	10	20.9 ± 17.1
Post	8	12	58	–	34	24	16	20	12	23.0 ± 16.4
FU	8	20	60	–	34	12	16	16	12	22.8 ± 17.0
**DHI**										
Pre	4	20	38	20	16	46	14	24	18	22.2 ± 12.7
Post	4	20	40	–	18	36	12	20	18	21.0 ± 11.8
FU	0	20	22	–	16	46	14	24	18	20.0 ± 12.8

DHI, Dizziness Handicap Inventory; f, female; freq, TN frequency; HHI, Hearing Handicap Inventory; int, TN intensity; m, male; N, non-tonal; pre, pre-operative TN onset; post, post-operative TN onset; T, tonal; TTI, Tinnitus Handicap Inventory;

* *drop out*.

### Clinical Evaluation

Hearing impairment was classified according to the Gardner & Robertson (GR) scale (34) based on the results of the pure tone audiometry (PTA) and speech discrimination (SDS) resulting in five classes: GR 1 (good, PTA 0–30 dB, and SDS 70–100%), GR 2 (serviceable, PTA 31–50 dB, and SDS 50–69%), GR 3 (non-serviceable, PTA 51–90 dB, and SDS 5–49%), GR 4 (poor, PTA 51–90 dB, and SDS 1–4%), GR 5 (deaf, PTA 0 dB, and SDS 0%). For statistical reasons, GR score was reclassified in (i) preserved hearing (GR 1–4) and (ii) no hearing (GR5). VS tumor size was graded according to the Hannover classification (5) into four classes: T1 (purely intrameatal), T2 (intra- and extrameatal), T3 (filling the cerebellopontine cistern), T4 (compressing the brain stem).

### Study Design

The aim of the study was to prove the feasibility and effectivity of repetitive transcranial magnetic stimulation (rTMS) therapy in a VS-associated TN. The study covered a treatment period of 10 consecutive workdays on which rTMS was applied. All patients received a standardized evaluation of VS-associated audiovestibular symptoms (i.e., hearing impairment, tinnitus, dizziness) with questionnaires (i.e., Hearing Handicap Inventory, HHI; Tinnitus Handicap Inventory, THI; Dizziness Handicap Inventory, DHI) at the first day (PRE) and the last day (POST) of the treatment period. Questionnaires were also acquired after 4 weeks (follow-up, FU) to evaluate the long-term effects. In addition, we evaluated the patient's daily TN perception with a Tinnitus Matching Test (TMT) just prior and after the rTMS application. The primary endpoint of the study was a reduction of distress suffered by the TN patient as measured by the THI. Secondary endpoint of the study was the reduction of TN perception as measured by the TMT and the evaluation of factors predicting the rTMS effect (i.e., hearing impairment, tinnitus duration, type of tinnitus.).

### Tinnitus Handicap Inventory

The Tinnitus Handicap Inventory (THI) is a self-administered test to determine the degree of distress suffered by the TN patient. The THI has been introduced in 1996 (35). It consists of 25 questions divided into three subgroups: functional, emotional, and catastrophic. Eleven items are included in the functional scale, nine in the emotional scale, and five in the catastrophic scale. A yes response yields a score of four points; sometimes, two points; and no, zero points. The total score ranges from zero (no disability) to 100 (severe disability). Studies have also indicated that the minimum change in the THI score that can be considered clinically relevant is a reduction of 6–7 points (35). It is widely used in medical offices and in clinical trials to determine the effectiveness of a given therapy (36, 37).

### Hearing Handicap Inventory and Dizziness Handicap Inventory

The HHI (38) and the DHI (39) are constructed equivalent to the THI and designed as self-administered 25-item questionnaires to determine the degree of disability in relation to hearing impairment and dizziness. A *yes* response yields a score of four points; *sometimes*, two points; and *no*, zero points. The total score ranges from zero (no disability) to 100 (severe disability). The scales consist of a 7-item physical subscale, a 9-item emotional subscale, and a 9-item functional subscale.

### Tinnitus Matching Test

The TMT was performed based on pure sinus waves (in tonal TN) or white gausian noise (wgn, in non-tonal/noise-like TN) provided by custom-written Matlab scripts and presented to both ears of the patient with headphones (HD4.30, Sennheiser, Wennebostel, Germany). In an iterative process, the patient was asked to provide feedback about the individual TN frequency (kHz, in tonal tinnitus) and TN intensity (in dB). Frequency and intensity were adjusted by the experimenter until the patient confirmed that the presented tone/noise matches to his/her individual TN. Frequency and/or intensity were noted for further analysis. The TMT was performed daily immediately prior and after the rTMS application. While TMT is an excellent way to objectify the subjective tinnitus sensation, it is subject to major problems which have been discussed elsewhere (40).

### TMS-Mapping and rTMS Parameter

T1-weighted MRI brain scans preceded the experiment to obtain individual anatomical images in combination with an e-field guided neuronavigational rTMS system (NBS, Nexstim, Finland). First, a single standard motor mapping of the right primary motor cortex was performed with a bipulse eight-figure coil (41–44). After determining the “hotspot” yielding the largest motor-evoked potential (MEP) from the left abductor pollicis brevis muscle (APB), the resting motor threshold (RMT), defined as the minimum stimulus intensity to result in at least 5/10 trials a MEP >50 μV, was obtained. The orientation of the induced current in the brain was posterior-anterior for the first phase and anterior-posterior for the second phase of the stimulus. The orientation of the electric field was kept perpendicular to the central sulcus. Subsequently, the cortex was mapped with 110% RMT starting at the primary motor cortex and then extending around this spot to cover the primary motor cortex, somatosensory cortex, and premotor cortex. The TMS coil was localized over the right DLPFC according to a standard algorithm by moving the coil from the APB hotspot 6 cm in the anterior direction (Figure 1C). The coordinates and direction of the e-field were saved and kept constant throughout the experiment. rTMS was applied with a bipulse eight-figure coil as a sequence of 1,200 pulses with a 1 Hz stimulation frequency and an intensity of 110% RMT (45).

### Statistical Analysis

All analysis and statistical tests were performed using MATLAB (MathWorks, Inc., Natick, MA, USA) and SPSS (IBM SPSS Statistics for Windows, Version 26.0. Armonk, NY: IBM Corp.). Significance of rTMS related changes in THI, HHI, and DHI were evaluated with paired *t*-test. Changes in the TN frequency and intensity over the course of the therapy were linearly fitted by the Matlab “robustfit.” To evaluate the impact of the hearing status (HEARING), TN duration (DURATION), and the TN type (TYPE) on the acute rTMS effect, an analysis of covariance was applied on the THI and the relative TN intensities. Pearson's regression analysis was performed to evaluate the correlation between the acute effect as measured by the THI and the TMT. Data are shown as the mean ± standard deviation (SD). *p* < 0.05 were considered significant. Finally, operative complications were evaluated, if detected in the postoperative CT scan.

## Results

**Figure 2:**
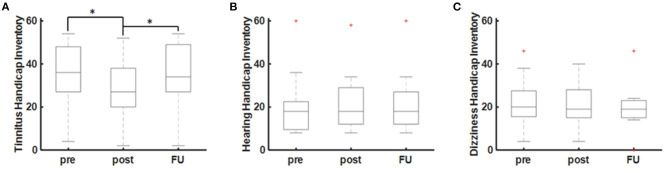
rTMS effects on audiovestibular symptoms. **(A)** Tinnitus as measured by the Tinnitus Handicap Inventory (THI). **(B)** Hearing as measured by the Hearing Handicap Inventory (HHI) and **(C)** dizziness as measured by the Dizziness Handicap Inventory (DHI). Statistical significance is marked by an asterisk (*p < 0.005; paired *t*-test).

There were no significant side effects of the rTMS stimulation in any of the patients. One patient (ID4) dropped-out of the study due to a subjective non-response to the treatment (see Table 1). There was a significant acute decrease of the THI scores at the end of the 10-day rTMS therapy (POST) in comparison to the baseline (PRE) values (*T* = 3.33, *p* = 0.013; paired *t*-test). The acute effect of rTMS on the THI values (PRE-POST) was approximated at −7.0 ± 6.0 [−16 0] points (*T* = −3.33, *p* = 0.013; one-sample *t*-test). As a reduction of 6–7 points in the THI are considered clinically relevant, only 4/9 (44.4%) are classified as good responders according to the THI. During the follow-up (FU) after 4 weeks, however, there was no sustained effect of rTMS (*T* = 0.683, *p* = 0.516; paired *t*-test) on the THI values (Figure 2A). In contrast, there was no short-term or long-term effect of rTMS on the HHI or DHI values (Figures 2B,C).

**Figure 3:**
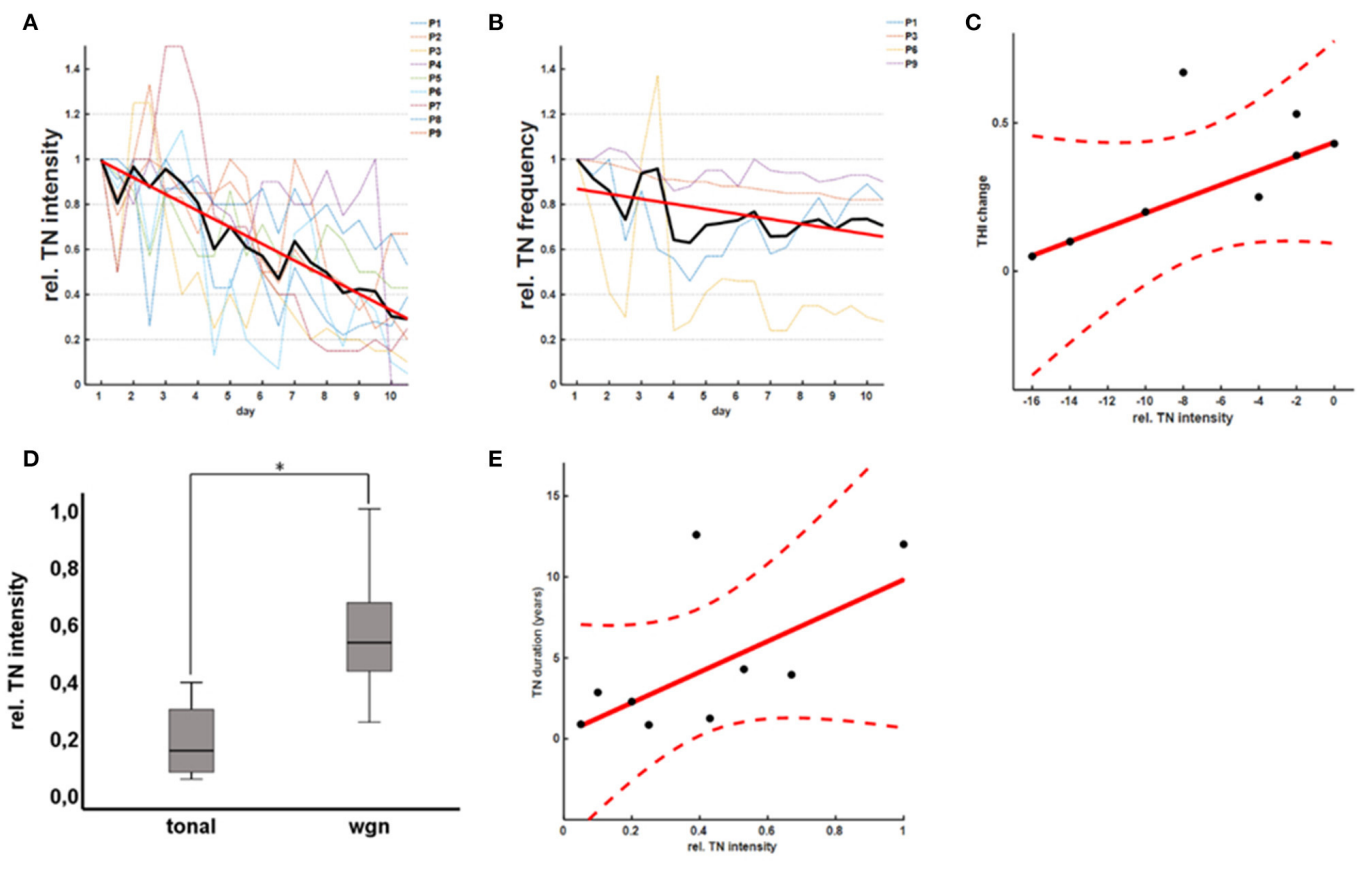
rTMS effects on TN matching. rTMS therapy led to a significant reduction of the TN intensity as measured by the TN matching **(A)**. In patients with a tonal TN, there was an additional tendency for a reduction of the TN frequency **(B)**. Notably, there was a good correlation between the acute effect as measured by the THI and the TN matching test **(C)**. In general, patients with a tonal TN **(D)** and shorter TN duration **(E)** showed a better response to rTMS therapy.

The acute effect of rTMS on tinnitus perception was confirmed by recordings of the TMT revealing a significant reduction of TN intensity (*b* = −0.068, *p* < 0.001; robust regression) over the course of the 10-day rTMS therapy (Figure 3A). The mean relative TN intensity after rTMS was 0.40 ± 0.30 [0.05–1.00] (*T* = −5.95, *p* < 0.001; one-sample *t*-test), indicating a reduction of TN intensity of ~60%. As a reduction of 20% is considered clinically relevant, 8/9 (89%) of the patients are classified as good responders according to the TMT. Moreover, 6/9 (67%) of the patients showed a reduction of TN intensity for more than 50%. For patients with tonal tinnitus (*n* = 4), there was a tendency for a slight reduction of TN frequency (*b* = −0.011, *p* = 0.009; robust regression) over the course of the 10-day rTMS therapy (Figure 3B). Notably, there was a good correlation (*r* = 0.43; Pearson's) between the acute effect as measured by the THI and the TMT (Figure 3C). To evaluate the effect of the hearing status (HEARING), TN duration (DURATION), and the TN type (TYPE) on the acute rTMS effect, an analysis of covariance was applied on the THI and the relative TN intensities at the end of the 10-day rTMS therapy. In fact, we could not detect any significant main effect of HEARING [*F*_(1, 4)_ = 1.56; *p* = 0.782], TYPE [*F*_(1, 4)_ = 4.01; *p* = 0.116], or DURATION [*F*_(1, 4)_ = 4.41; *p* = 0.104] on the TN perception as measured by the THI questionnaire. Considering the TN matching results, however, we observed a significant effect of TYPE [F_(1, 4)_ = 10.29; *p* = 0.024] and DURATION [F_(1, 4)_ = 17.81; *p* = 0.008]. Patients with a tonal TN showed a higher benefit from therapy than patients with a noise-like TN (Figure 3D). Additionally, patients with a longer TN duration showed less TN intensity reduction after rTMS (Figure 3E). Basically, patients suffering from TN for <5 years showed a better response than patients suffering from tinnitus for more than 10 years. In contrast, there was no effect of HEARING [*F*_(1, 4)_ = 0.25; *p* = 0.637].

## Discussion

The present pilot study proves a significant acute effect of low-frequency rTMS to the right DLPFC on TN perception (THI and TMT) of nine VS patients. However, there was no significant long-term effect in the follow-up after 4 weeks. In contrast, rTMS had no effect on other audiovestibular complaints such as hearing impairment or dizziness. While the THI failed to detect any clinically relevant acute effect of rTMS in 56% of the patients, TMT revealed a reduction of TN intensity for more than 20 in 89% and for more than 50 in 56% of the patients. Notably, the acute effect of rTMS was influenced by the TN type and duration. In general, patients with a tonal TN and shorter TN duration showed a better response to rTMS therapy.

Considering the hypothesized pathophysiology of TN driven by a central maladaptation, rTMS has been suggested in TN treatment several years ago. However, the results have been divergent and even contradictory (21, 24, 26–29). rTMS applies a train of repetitive magnetic pulses to alter the excitability of the neurons and modulate cortical activity. High-frequency rTMS increases cortical excitability, while low-frequency rTMS is considered to inhibit the neural activity in stimulated regions (46, 47). In general, rTMS has been successfully applied for TN treatment at the primary auditory cortex (36, 37), temporoparietal junction (48), and dorsolateral pre-frontal cortex (DLPFC) (45, 49). Low-frequency rTMS to temporal stimulation sites is supposed to reduce the hyperexcitability of the auditory network (36, 37). However, a recent multicenter randomized controlled trial with a large sample size demonstrated that low-frequency (1 Hz) rTMS over the temporal cortex is not superior to sham rTMS in reducing TN severity (37). To the authors' opinion, the temporal lobe is not an optimal target candidate for rTMS application due to its coverage by the temporal muscle. Depending on the individual's anatomy, the temporal muscle might increase the distance to the cortex and thus, potentially decrease the strength of the magnetic field on the cortex to subthreshold levels. Additionally, involuntary muscle twitches related to the TMS pulses are limiting the applicable stimulation intensity. In contrast, stimulation to the DLPFC is easily accessible and thought to influence networks involved in auditory attention (50). Interestingly, TN suppression has been shown for either *high*-frequency stimulation of the *left* DLPFC (49) and *low*-frequency stimulation of the *right* DLPFC (45). Furthermore, combined multisite rTMS is hypothesized to improve treatment outcome (48). In a recent meta-analysis, however, concurrent high-frequency stimulation of the *left* DLPFC to the temporal cortex was not found to promote efficacy (23). Considering this evidence, we have decided on the application of *low*-frequency stimulation of the *right* DLPFC (45) for TN treatment. In fact, the present study represents the first study applying *low*-frequency rTMS on the *right* DLPFC in VS-associated TN.

Although being a more homogeneous patient cohort with a shorter TN duration than other published data (26), our response rates were quite comparable to other studies ranging around 50% of the treated patients (51). The application of rTMS in VS patients did not improve the treatment outcome substantially. However, the number of treated patients in this study is too low to draw final conclusions. Additionally, the sensitivity of the THI questionnaire might be—although widely used (21, 36, 37)—too low to detect slight rTMS-related TN improvements (52, 53). In contrast, there was a clear effect of rTMS on the TMT values. We hypothesize that the treatment duration might be too short to induce a clinically relevant effect measurable by the THI. In depression treatment, rTMS exerts a positive clinical effect after four rather than 2 weeks of treatment (54–56).

Notably, our findings indicate that patients with a tonal TN and shorter TN duration showed a better response to rTMS therapy. These findings are in good accordance with the previous publication indicating an impact of these factors on the treatment outcome (30–33). In particular, tonal TN in comparison to non-tonal/noise-like TN is suggested to benefit from rTMS therapy (32, 33). In contrast to the other studies showing an effect of hearing impairment on the treatment outcome (30, 31), our findings did not reproduce these observations. Due to statistical reasons, we have dichotomized hearing impairment in the present study, which might mask the actual effect. However, the number of patients is too low for more profound statistical evaluation. Finally, although our study indicates an acute rTMS effect on VS-associated TN, there is no evidence for a long-term effect after a period of 4 weeks (21, 24, 26–29). However, independent of the sustained long-term effect, rTMS might be useful for priming the cortex in chronic TN patients in order to increase the susceptibility for further treatment options, e.g., notched-noise therapy, pharmacological intervention, cognitive behavioral therapy, TN masking, or music therapy (10, 11). Finally, there is an increasing literature indicating that TMS effects depend on the ongoing brain-state (57, 58). Thus, applying TMS in VS treatment in a closed-loop fashion depending on the ongoing brain state might improve the treatment outcome. Comparable observations have been made in the recruitment of additional corticospinal tracts (41, 59) for e.g., stroke therapy. In line with this, a dependency of rTMS-based TN therapy on cortical alpha oscillations has been described recently (60, 61).

### Limitations of the Study

There are several limitations to the present study. First, the number of patients is too low to draw final conclusions about the effectivity of rTMS in VS-associated patients. However, our findings are comparable to the known results in literature.Second, there was no control group.Interventional studies should ideally be designed as randomized, double-blinded controlled studies (36, 37). However, considering the low incidence of VS-associated TN in comparison to other types of TN, it will be difficult to achieve an adequate sample size.

## Conclusion

The present pilot study is the first one exclusively evaluating the effect of rTMS in VS-associated TN. Our results prove the feasibility and the efficacy of low-frequency rTMS to the right DLPFC in this patient cohort. The results were comparable to the available data with a significant acute but limited long-term effect. However, there is evidence that patients with a tonal TN and shorter TN duration might have the strongest benefit. A randomized controlled study with a larger sample size is necessary to prove these initial findings.

## Data Availability Statement

The raw data supporting the conclusions of this article will be made available by the authors, without undue reservation.

## Ethics Statement

The studies involving human participants were reviewed and approved by Ethics committee of the Eberhard Karls University Tuebingen. The patients/participants provided their written informed consent to participate in this study.

## Author Contributions

MTL and GN were responsible for data acquisition, data analyses, statistical analysis as well as drafting and reviewing the manuscript. ML, JS, and KM were involved in data acquisition as well as drafting and reviewing the manuscript. MT and AS were involved in drafting and reviewing the manuscript. All authors contributed to the article and approved the submitted version.

## Conflict of Interest

The authors declare that the research was conducted in the absence of any commercial or financial relationships that could be construed as a potential conflict of interest.

## Abbreviations

DHI: Dizziness Handicap inventory
DLPFC: dorsolateral pre-frontal cortex
FU: follow-up
GR: Gardner & Robertson scale
HHI: Hearing Handicap Inventory
PTA: pure tone audiometry
RMT: resting motor threshold
rTMS: repetitive transcranial magnetic stimulation
SDS: speech discrimination
THI: Tinnitus Handicap inventory
TMT: tinnitus matching test
TN: tinnitus
VS: vestibular schwannoma.

## References

[1] Baguley DM Humphriss RL Axon PR Moffat DA. The clinical characteristics of tinnitus in patients with vestibular schwannoma. Skull Base. (2006) 16:49–58. 10.1055/s-2005-926216 17077869 PMC1502033

[2] Lloyd SKW Kasbekar AV Baguley DM Moffat DA. Audiovestibular factors influencing quality of life in patients with conservatively managed sporadic vestibular schwannoma. Otol Neurotol. (2010) 31:968–76. 10.1097/MAO.0b013e3181e8c7cb 20684063

[3] Naros G Sandritter J Liebsch M Ofori A Rizk AR Del Moro G. Predictors of preoperative tinnitus in unilateral sporadic vestibular schwannoma. Front Neurol. (2017) 8:378. 10.3389/fneur.2017.00378 28824535 PMC5541055

[4] Trakolis L Ebner FH Machetanz K Sandritter J Tatagiba M Naros G. Postoperative tinnitus after vestibular schwannoma surgery depends on preoperative tinnitus and both pre- and postoperative hearing function. Front Neurol. (2018) 9:136. 10.3389/fneur.2018.00136 29593635 PMC5857542

[5] Matthies C Samii M. Management of 1000 vestibular schwannomas (acoustic neuromas): clinical presentation. Neurosurgery. (1997) 40:1–9; discussion 9–10. 10.1227/00006123-199701000-00001 8971818

[6] Moffat DA Baguley DM Beynon GJ Da Cruz M. Clinical acumen and vestibular schwannoma. Am J Otol. (1998) 19:82–7. 9455955

[7] Myrseth E Pedersen PH Møller P Lund-Johansen M. Treatment of vestibular schwannomas. Why, when and how? Acta Neurochir. (2007) 149:647–60; discussion 660. 10.1007/s00701-007-1179-0 17558460

[8] Del Río L Lassaletta L Díaz-Anadón A Alfonso C Roda JM Gavilán J. Tinnitus and quality of life following vestibular schwannoma surgery. B-ENT. (2012) 8:167–71. Available online at: http://www.b-ent.be/en/tinnitus-and-quality-of-life-following-vestibular-schwannoma-surgery-13446 23113378

[9] Grauvogel J Kaminsky J Rosahl SK. The impact of tinnitus and vertigo on patient-perceived quality of life after cerebellopontine angle surgery. Neurosurgery. (2010) 67:601–9. 10.1227/01.NEU.0000374725.19259.EA 20647966

[10] Baguley D McFerran D Hall D. Tinnitus. Lancet. (2013) 382:1600–7. 10.1016/S0140-6736(13)60142-7 23827090

[11] Langguth B Kreuzer PM Kleinjung T De Ridder D. Tinnitus: causes and clinical management. Lancet Neurol. (2013) 12:920–30. 10.1016/S1474-4422(13)70160-1 23948178

[12] Chovanec M Zvěrina E Profant O Balogová Z Kluh J Syka J. Does attempt at hearing preservation microsurgery of vestibular schwannoma affect postoperative tinnitus? Biomed Res. Int. (2015) 2015:783169. 10.1155/2015/783169 25654125 PMC4309247

[13] Kohno M Shinogami M Yoneyama H Nagata O Sora S Sato H. Prognosis of tinnitus after acoustic neuroma surgery–surgical management of postoperative tinnitus. World Neurosurg. (2014) 81:357–67. 10.1016/j.wneu.2012.09.008 23022637

[14] Han BI Lee HW Kim TY Lim JS Shin KS. Tinnitus: characteristics, causes, mechanisms, and treatments. J Clin Neurol. (2009) 5:11. 10.3988/jcn.2009.5.1.11 19513328 PMC2686891

[15] Møller AR. Pathophysiology of tinnitus. Otolaryngol Clin North Am. (2003) 36:249–66, v–vi. 10.1016/S0030-6665(02)00170-6 12856295

[16] O'Connor AF France MW Morrison AW. Perilymph total protein levels associated with cerebellopontine angle lesions. Am J Otol. (1981) 2:193–5. 6974501

[17] Sahley T Nodar R Musiek F Sahley TL Nodar RH Musiek FE. Efferent Auditory System: Structure and Function, Singular Publishing Group Audiology series. San Diego, CA: Singular Publishing Group (1997).

[18] Henry JA Roberts LE Caspary DM Theodoroff SM Salvi RJ. Underlying mechanisms of tinnitus: review and clinical implications. J Am Acad Audiol. (2014) 25:5–22; quiz 126. 10.3766/jaaa.25.1.2 24622858 PMC5063499

[19] Shore SE Roberts LE Langguth B. Maladaptive plasticity in tinnitus–triggers, mechanisms and treatment. Nat Rev Neurol. (2016) 12:150–60. 10.1038/nrneurol.2016.12 26868680 PMC4895692

[20] Langguth B. Non-invasive neuromodulation for tinnitus. J Audiol Otol. (2020) 24:113–8. 10.7874/jao.2020.00052 32575951 PMC7364190

[21] Dong C Chen C Wang T Gao C Wang Y Guan X. Low-Frequency repetitive transcranial magnetic stimulation for the treatment of chronic tinnitus: a systematic review and meta-analysis of randomized controlled trials. Biomed Res Int. (2020) 2020:3141278. 10.1155/2020/3141278 32461976 PMC7218966

[22] Schoisswohl S Arnds J Schecklmann M Langguth B Schlee W Neff P. Amplitude modulated noise for tinnitus suppression in tonal and noise-like tinnitus. Audiol Neurotol. (2020) 24:309–21. 10.1159/000504593 PMC6959056 31905364

[23] Schoisswohl S Agrawal K Simoes J Neff P Schlee W Langguth B. RTMS parameters in tinnitus trials: a systematic review. Sci Rep. (2019) 9:12190. 10.1038/s41598-019-48750-9 31434985 PMC6704094

[24] Theodoroff SM Folmer RL. Repetitive transcranial magnetic stimulation as a treatment for chronic tinnitus: a critical review. Otol Neurotol. (2013) 34:199–208. 10.1097/MAO.0b013e31827b4d46 23444467

[25] May A Hajak G Gänßbauer S Steffens T Langguth B Kleinjung T. Structural brain alterations following 5 days of intervention: dynamic aspects of neuroplasticity. Cereb Cortex. (2007) 17:205–10. 10.1093/cercor/bhj138 16481564

[26] Liang Z Yang H Cheng G Huang L Zhang T Jia H. Repetitive transcranial magnetic stimulation on chronic tinnitus: a systematic review and meta-analysis. BMC Psychiatry. (2020) 20:547. 10.1186/s12888-020-02947-9 33228598 PMC7684956

[27] Meng Z Liu S Zheng Y Phillips JS. Repetitive transcranial magnetic stimulation for tinnitus. Cochrane Database Syst Rev. (2011) 5:CD007946. 10.1002/14651858.CD007946.pub2 21975776

[28] Peng Z Chen XQ Gong SS. Effectiveness of repetitive transcranial magnetic stimulation for chronic tinnitus: a systematic review. Otolaryngol Head Neck Surg. (2012) 147:817–25. 10.1177/0194599812458771 22941756

[29] Soleimani R Jalali MM Hasandokht T. Therapeutic impact of repetitive transcranial magnetic stimulation (rTMS) on tinnitus: a systematic review and meta-analysis. Eur Arch Oto-Rhino Laryngol. (2016) 273:1663–75. 10.1007/s00405-015-3642-5 25968009

[30] De Ridder D Verstraeten E Van Der Kelen K De Mulder G Sunaert S Verlooy J. Transcranial magnetic stimulation for tinnitus: Influence of tinnitus duration on stimulation parameter choice and maximal tinnitus suppression. Otol Neurotol. (2005) 26:616–9. 10.1097/01.mao.0000178146.91139.3c 16015156

[31] Kleinjung T Steffens T Sand P Murthum T Hajak G Strutz J. Which tinnitus patients benefit from transcranial magnetic stimulation? Otolaryngol Head Neck Surg. (2007) 137:589–95. 10.1016/j.otohns.2006.12.007 17903575

[32] Astrid L Martin S Michael L Michael KP Timm BP Elmar F. Predictors for rTMS response in chronic tinnitus. Front Syst Neurosci. (2012) 6:11. 10.3389/fnsys.2012.00011 22383902 PMC3284861

[33] Frank G Kleinjung T Landgrebe M Vielsmeier V Steffenhagen C Burger J. Left temporal low-frequency rTMS for the treatment of tinnitus: clinical predictors of treatment outcome-a retrospective study. Eur J Neurol. (2010) 17:951–6. 10.1111/j.1468-1331.2010.02956.x 20158510

[34] Gardner G Robertson JH. Hearing preservation in unilateral acoustic neuroma surgery. Ann Otol Rhinol Laryngol. (1988) 97:55–66. 10.1177/000348948809700110 3277525

[35] McCombe A Baguley D Coles R McKenna L McKinney C Windle-Taylor P. Guidelines for the grading of tinnitus severity: the results of a working group commissioned by the British association of otolaryngologists, head and neck surgeons, 1999. Clin Otolaryngol Allied Sci. (2001) 26:388–93. 10.1046/j.1365-2273.2001.00490.x 11678946

[36] Folmer RL Theodoroff SM Casiana L Shi Y Griest S Vachhani J. Repetitive transcranial magnetic stimulation treatment for chronic tinnitus: a randomized clinical trial. JAMA Otolaryngol Head Neck Surg. (2015) 141:716–22. 10.1001/jamaoto.2015.1219 26181507

[37] Landgrebe M Hajak G Wolf S Padberg F Klupp P Fallgatter AJ. 1-Hz rTMS in the treatment of tinnitus: a sham-controlled, randomized multicenter trial. Brain Stimul. (2017) 10:1112–20. 10.1016/j.brs.2017.08.001 28807845

[38] Newman CW Weinstein BE Jacobson GP Hug GA. Test-retest reliability of the hearing handicap inventory for adults. Ear Hear. (1991) 12:355–7. 10.1097/00003446-199110000-00009 1783240

[39] Kurre A Van Gool CJAW Bastiaenen CHG Gloor-Juzi T Straumann D De Bruin ED. Translation, cross-cultural adaptation and reliability of the German version of the dizziness handicap inventory. Otol Neurotol. (2009) 30:359–67. 10.1097/MAO.0b013e3181977e09 19225437

[40] Klouse KP. Measurement procedures. US Bur Mines Inf Circ. (1975) 1:10–15.

[41] Kraus D Naros G Bauer R Leão MT Ziemann U Gharabaghi A. Brain-robot interface driven plasticity: distributed modulation of corticospinal excitability. Neuroimage. (2016) 125:522–32. 10.1016/j.neuroimage.2015.09.074 26505298

[42] Kraus D Gharabaghi A. Projecting navigated TMS sites on the gyral anatomy decreases inter-subject variability of cortical motor maps. Brain Stimul. (2015) 8:831–7. 10.1016/j.brs.2015.03.006 25865772

[43] Leão MT Naros G Gharabaghi A. Detecting poststroke cortical motor maps with biphasic single- and monophasic paired-pulse TMS. Brain Stimul. (2020) 13:1102–4. 10.1016/j.brs.2020.05.005 32418913

[44] Mathew J Kübler A Bauer R Gharabaghi A. Probing corticospinal recruitment patterns and functional synergies with transcranial magnetic stimulation. Front Cell Neurosci. (2016) 10:175. 10.3389/fncel.2016.00175 27458344 PMC4932869

[45] De Ridder D Song JJ Vanneste S. Frontal cortex TMS for tinnitus. Brain Stimul. (2013) 6:355–62. 10.1016/j.brs.2012.07.002 22853891

[46] Hallett M. Transcranial magnetic stimulation: a primer. Neuron. (2007) 55:187–99. 10.1016/j.neuron.2007.06.026 17640522

[47] Hallett M. Transcranial magnetic stimulation and the human brain. Nature. (2000) 406:147–50. 10.1038/35018000 10910346

[48] Piccirillo JF Kallogjeri D Nicklaus J Wineland A Spitznagel EL Vlassenko AG. Low-frequency repetitive transcranial magnetic stimulation to the temporoparietal junction for tinnitus: four-week stimulation trial. JAMA Otolaryngol Head Neck Surg. (2013) 139:388–95. 10.1001/jamaoto.2013.233 23599075 PMC4605206

[49] Vanneste S De Ridder D. The involvement of the left ventrolateral prefrontal cortex in tinnitus: a TMS study. Exp Brain Res. (2012) 221:345–50. 10.1007/s00221-012-3177-6 22782483

[50] Vanneste S De Ridder D. The auditory and non-auditory brain areas involved in tinnitus. An emergent property of multiple parallel overlapping subnetworks. Front Syst Neurosci. (2012) 6:31. 10.3389/fnsys.2012.00031 22586375 PMC3347475

[51] Dornhoffer JL Mennemeier M. Using repetitive transcranial magnetic stimulation for the treatment of tinnitus. Hear J. (2010) 63:16–20. 10.1097/01.HJ.0000390816.71876.aa 24647744 PMC3955991

[52] Kamalski DM Hoekstra CE Van Zanten BG Grolman W Rovers MM. Measuring disease-specific health-related quality of life to evaluate treatment outcomes in tinnitus patients: a systematic review. Otolaryngol Head Neck Surg. (2010) 143:181–5. 10.1016/j.otohns.2010.03.026 20647116

[53] Meikle MB Henry JA Griest SE Stewart BJ Abrams HB McArdle R. The tinnitus functional index: development of a new clinical measure for chronic, intrusive tinnitus. Ear Hear. (2012) 33:153–76. 10.1097/AUD.0b013e31822f67c0 22156949

[54] Levkovitz Y Isserles M Padberg F Lisanby SH Bystritsky A Xia G. Efficacy and safety of deep transcranial magnetic stimulation for major depression: a prospective multicenter randomized controlled trial. World Psychiatry. (2015) 14:64–73. 10.1002/wps.20199 25655160 PMC4329899

[55] Loo CK Mitchell PB. A review of the efficacy of transcranial magnetic stimulation (TMS) treatment for depression, and current and future strategies to optimize efficacy. J Affect Disord. (2005) 88:255–67. 10.1016/j.jad.2005.08.001 16139895

[56] O'Reardon JP Solvason HB Janicak PG Sampson S Isenberg KE Nahas Z. Efficacy and safety of transcranial magnetic stimulation in the acute treatment of major depression: a multisite randomized controlled trial. Biol Psychiatry. (2007) 62:1208–16. 10.1016/j.biopsych.2007.01.018 17573044

[57] Khademi F Royter V Gharabaghi A. Distinct beta-band oscillatory circuits underlie corticospinal gain modulation. Cereb Cortex. (2018) 28:1502–15. 10.1093/cercor/bhy016 29415124 PMC6093341

[58] Naros G Lehnertz T Leão MT Ziemann U Gharabaghi A. Brain state-dependent gain modulation of corticospinal output in the active motor system. Cereb Cortex. (2020) 30:371–81. 10.1093/cercor/bhz093 31204431

[59] Kraus D Naros G Guggenberger R Leão MTMT Ziemann U Gharabaghi A. Recruitment of additional corticospinal pathways in the human brain with state-dependent paired associative stimulation. J Neurosci. (2018) 38:1396–407. 10.1523/JNEUROSCI.2893-17.2017 29335359 PMC6705844

[60] Müller N Lorenz I Langguth B Weisz N. rTMS induced tinnitus relief is related to an increase in auditory cortical alpha activity. PLoS ONE. (2013) 8:e55557 10.1371/journal.pone.0055557 23390539 PMC3563643

[61] Weisz N Lüchinger C Thut G Müller N. Effects of individual alpha rTMS applied to the auditory cortex and its implications for the treatment of chronic tinnitus. Hum Brain Mapp. (2014) 35:14–29. 10.1002/hbm.22152 23008160 PMC6869412

